# Comparative study of personality disorder associated with deliberate self harm in two different age groups (15–24 years and 45–74 years)

**DOI:** 10.4103/0019-5545.43630

**Published:** 2008

**Authors:** Saswati Nath, Dipak Kumar Patra, Srilekha Biswas, Asim Kumar Mallick, Gautam Kumar Bandyopadhyay, Srijit Ghosh

**Affiliations:** Department of Psychiatry, R.G.Kar Medical College and Hospital, Kolkata, India; 1 Department of Pediatrics, Medical College, Kolkata, India; 2 Institute of Psychiatry, N.R.S. Medical College, Kolkata, India; 3 Department of Psychiatry, N.R.S. Medical College, Kolkata, India

**Keywords:** Deliberate self harm, personality disorder, suicide

## Abstract

**Aims:**

To study the presence of personality disorder in cases of deliberate self harm (DSH) in young (15–24 years) and elderly (45–74 years) and compare.

**Materials and Methods:**

Deliberate self harm cases admitted in Medical and surgical departments and cases attending psychiatry department of R.G. Kar Medical College, Kolkata were studied. For diagnosis of personality disorder ICD 10 International Personality Disorder Examination (IPDE) questionnaire was used.

**Results:**

Percentage of elderly patients having personality disorder (64%) was higher compared to young DSH patients (58.5%). In young group, most common disorder was emotionally unstable personality disorder (28.6%) and in elderly group most common was anankastic type of personality disorder (36%). Schizoid, dissocial, histrionic, and anxious-avoidant personality disorders were found in small percentages of cases.

**Conclusion:**

Among DSH patients, the most common personality disorder found in young age was Emotionally unstable (Impulsive and Borderline) personality disorder, but most common personality disorder found in elderly patients was Anankastic personality disorder.

## INTRODUCTION

The prevention of suicide is a national and international policy priority.[1] Personality disorders and their co-morbidity with other psychiatric conditions are risk factors for both fatal and nonfatal suicidal behaviors, and self-mutilation,[2] Personality of a person determines how a person will react in a stressful situation.

15-24 years age group is selected as from previous study reports it is seen that the rate of deliberate self-harm (DSH) is rising in the age group 15–24 years and 35–45 years.[3] Old age is an important predictor of completed suicide[1] as the number of patients of DSH in old age is less, so larger age range is taken.

Risk of successful suicide after an attempt is greater in older patients, who are male, depressed or alcoholic.[4] But unlike the active, impulsive suicide attempts often seen in younger population, late life suicide is more likely to be passive, premeditated and lethal. Rates of suicide in elderly may be even higher than those reported.

Predisposing factors for suicide and attempted suicide also differs in early and old age. Late life is often marked by social isolation, stressful events and profound losses (e.g. spouse, family member, friends, home and health). Many studies have found that both physical and psychiatric illnesses are correlated with increased rates of suicide among elderly.

But there are very few studies regarding prevalence of different types of personality disorder in DSH patients, especially in elderly people. Our aim is to see the type of personality disorder most vulnerable to self-harm activity and whether it differs in young and elderly people.

Here we have used a rating scale (IPDE) for assessment of personality disorder.

### Aims and objectives

1. To study the type of personality disorder commonly associated with DSH.
2. To compare prevalence of different types of personality disorders between young and elderly people.

## MATERIALS AND METHODS

### Place of study

In-patient department of Medicine and Surgery and out patient department (OPD) of Psychiatry in R.G. Kar Medical College and Hospital.

### Study population

Cases of DSH of age groups (a) 15–24 years and (b) 45–74 years who fulfilled the inclusion and exclusion criteria.

#### Inclusion criteria

- Must have a history of at least one DSH in their lifetime.
- Must be able to answer to questions.

#### Exclusion criteria

- Individuals beyond the age groups 15–24 years and 45–74 years.
- Patients who are seriously ill, physically or mentally.

### Sampling strategy

During the study period consecutive DSH patients that satisfied the inclusion and exclusion criteria have been taken into study.

### Sample size

#### Total – 106 patients

In 15–24 years age group – 77 patients

In 45–74 years age group – 29 patients

### Duration of study: Six months

#### Tools used

- A case history proforma
- ICD10 International Personality Disorder Examination (IPDE) questionnaire.

### Method

Cases of DSH admitted in Medical and Surgical Departments of R.G. Kar Medical College and Hospital and patients attending psychiatry OPD with history of at least one DSH in lifetime and within the age groups 15–24 years and 45–74 years were studied. Patients who are seriously ill or have severe mental disturbance were excluded from the study group. Indoor patients were traced from emergency admission register. Detail histories of all patients were taken from patients and family members.

To study personality disorder, ICD 10 International Personality Disorder Examination (IPDE) questionnaire was used. Patients who came to psychiatry OPD were interviewed there only. Among the patients admitted in indoor, those who were physically and mentally stable were interviewed in indoor taking them in a separate room. But the patients who were ill because of self harm act were advised to come to psychiatry OPD for follow up. Patients who came for follow up were interviewed in psychiatry OPD. All patients were interviewed. So, screening questionnaire was not used. Questions were explained to them in local language. It took about one hour for each patient. Some patients were a little resistant to talk because of concern about family members as they were all under police case file. But when the purpose was explained to them, none of them refused to respond to IPDE. Answers were recorded in answer sheet and scoring was done according to score sheet. Diagnosis of personality disorder came out from score sheet.

**Statistical analyses** – Z test for proportion.

## RESULTS

Personality disorder was found in 45 (58.5%) of young and 18 (62.1%) of elderly persons. Emotionally unstable (impulsive, borderline or both):

In young age group – 28.6%, in elderly people – 13.8%.

Anankastic Personality disorder:

Young – 11.7%, Elderly – 34.5%[Table 1].

**Table 1:** Distribution of personality disorder in dsh patients

Personality disorder	15–24 years			45–74 years		
		
	Male	Female	Total	Male	Female	Total
No definite disorder	7 (43.8)	25 (40.9)	32 (41.5)	7 (36.8)	4 (40)	11 (37.9)
F 60.0	0	2	2	1	1	2
Paranoid	(0)	(3.3)	(2.6)	(5.3)	(10)	(6.9)
F 60.1	0	0	0	0	0	0
Schizoid (0)	(0)	(0)	(0)	(0)	(0)	(0)
F 60.2	0	1	1	0	0	0
Dissocial	(0)	(1.6)	(1.3)	(0)	(0)	(0)
F 60.3	4	18	22	3	1	4
Emotionally unstable (impulsive, borderline or both)	(25)	(29.5)	(28.6)	(15.8)	(10)	(13.8)
F 60.4	0	1	1	0	0	0
Histrionic	(0)	(1.6)	(1.3)	(0)	(0)	(0)
F 60.5	3	6	9	7	3	10
Anankastic	(18.8)	(9.8)	(11.7)	(36.8)	(30)	(34.5)
F 60.6	0	2	2	0	0	0
Anxious avoidant	(0)	(3.3)	(2.6)	(0)	(0)	(0)
F 60.7	0	1	1	0	0	0
Dependent	(0)	(1.6)	(1.3)	(0)	(0)	(0)
F 60.9	1	2	3	1	1	2
Unspecified	(6.2)	(3.3)	(3.9)	(5.3)	(10)	(6.9)
More than one	1	3	4	0	0	0
Disorder	(6.2)	(4.9)	(5.2)	(0)	(0)	(0)
Total	16	61	77	19	10	29
	(100)	(100)	(100)	(100)	(100)	(100)

Figures in the parentheses indicate percentages.

## DISCUSSION

In this study, our aim was to compare the type of personality disorder commonly associated with DSH in young and elderly people. Personality disorder was found in 45 (58.5%) of young and 18 (62.1%) of elderly persons. Haw C *et al.* in 2001 found personality disorder in 45.9% of patients in general hospital in UK.[5] Equivalent proportions were also found by Haw C *et al.* in 2001.[5] We got higher proportion of personality disorder in elderly patients. This was not seen in previous studies. Our study was a time-bound study and we got very less number of patients in elderly group. To clarify the fact, study with more number of elderly patients has to be done.

In our study, dominant disorder differed in the two age groups. Interesting differences were also found in males and females of the two age groups.

Commonest type of personality disorder found in young age was emotionally unstable (impulsive, borderline or both) – 28.6%. But, it was comparatively less in elderly people (13.8%). This difference is statistically not significant at 5% level.

In case of elderly people, most common type was anankastic personality disorder – 34.5%, which was seen less commonly in young people (11.7%). This difference of proportion is statistically significant at 5% level.

In case of young people, emotionally unstable personality disorder was more common in females than in males (29.5% vs. 25%), but, in case of elderly it was more common in males (15.8% vs. 10%).

In both groups, anankastic personality disorder was found more commonly in males. Small percentages of patients in both groups had paranoid and unspecified type of personality disorder.

Most of the previous studies reported borderline personality disorder as the most common type in DSH patients including all ages. Yen S *et al*.[6] found borderline personality disorder in 79% of patients with suicidal behavior (no suicidal intent) and 78% of patients with attempted suicide. But in studies by Haw C *et al.* in 2001[5] personality types found were anxious (20.7%), anankastic (19.8%) and paranoid type (15.3%) and borderline in 10.8%. They found no gender difference and they included patients of all ages.

In our study, during interview it was evident from the statement of young patients that impulsive emotional outbursts were the causes of DSH in most of the cases without any long-standing problem and in many cases there was no suicidal intent. It was just to get an immediate relief or to manipulate a situation.

But in old age, in majority of cases, inability of obsessive persons to cope with the changing lifestyles of their younger generation was the cause of suicidal attempts. It was the statement of many elderly patients that their family members refused to abide by their rules and regulations and that led to frequent tension and discord. For obsessive persons, since ideology and correctness is placed before love and loyalty, divisiveness can break familial ties.[7]

As reported by Davis C and Karvinen K,[8] obsessive-compulsive symptoms and addictive personality characteristics were greater in those with an urge to self-harm. They also said that, impulsivity and compulsivity, both are more prominent in those with an intention to self-harm.

Another factor that may lead to suicide attempt in obsessive persons is inability to live up to their own expected correct standard. As stated by Steven Philipson, ‘since one's Humanness prevents an obssesive compulsive personality disorder sufferer from living according his own high standards, a tremendous amount of self-hatred is imposed’.[7]

## CONCLUSION

Results of the above study can be summarized in the following way:

- About half of the people in both groups were found to have personality disorder. Prevalence of personality disorder was more in elderly patients.
- Dominant personality disorder found in young patients was emotionally unstable personality disorder, but dominant disorder in elderly patients was anankastic personality disorder.
- In young patients, emotionally unstable personality disorder was found more commonly in females than in males, but in elderly patients it was more common in males.
- In both groups, anankastic personality disorder was found more commonly in males.
- 5.2% of young and none of elderly patients satisfied criteria for more than one disorder.

As the majority of studies conducted to date have concentrated on borderline personality disorder and antisocial personality disorder, the prevalence of DSH in other personality disorders requires further clarification.
